# Impact of COVID-19 on Clinical Practices during Lockdown: A pan India Survey of Orthopaedic Surgeons

**DOI:** 10.5704/MOJ.2103.009

**Published:** 2021-03

**Authors:** VK Jain, GK Upadhyaya, KP Iyengar, MK Patralekh, H Lal, R Vaishya

**Affiliations:** 1 Department of Orthopaedics, Dr Ram Manohar Lohia Hospital, New Delhi, India; 2 Department of Orthopaedics, All India Institute of Medical Sciences, Raibarelly, India; 3 Department of Orthopaedics, Southport and Ormskirk Hospital NHS Trust, Southport, United Kingdom; 4 Department of Orthopaedics, Vardhman Mahavir Medical College and Safdarjung Hospital, New Delhi, India; 5 Department of Orthopaedics, Indraprastha Apollo Hospital, New Delhi, IndiaDepartment of Orthopaedics, Vardhman Mahavir Medical College and Safdarjung Hospital, New Delhi, India

**Keywords:** COVID-19, coronavirus, pandemics, orthopaedics, orthopaedic procedures

## Abstract

**Introduction:**

The social lockdown measures imposed to contain the COVID-19 pandemic, have had profound effects on the healthcare systems across the world and India has been no exception to it. The study was aimed to evaluate the impact of COVID-19 on orthopaedic practice in India during the lockdown period and assess the preparedness of orthopaedic surgeons for resuming clinical practice after the initial lockdown was lifted.

**Materials and Methods:**

An online survey of 35 questions was conducted to evaluate impact on (i) general orthopaedic practice (ii) hospital protocols (iii) out-patient practice (iv) surgical practice (v) personal protective equipment (PPE) use and (vi) post-lockdown preparedness.

**Results:**

A total number of 588 practising orthopaedic surgeons from India completed the survey. Majority (88.3%) found severe impact (>50%) on trauma surgery and non-trauma surgery with significant reduction in out -patient attendance compared to corresponding time in 2019. There were significant changes made in individual hospital protocols (91.7 %). Appropriate required PPE was available in majority of the hospitals (74.3%). No remodelling or upgrading of the existing operating theatre infrastructure was done by most surgeons (89.5%).

**Conclusion:**

This pan India survey of orthopaedic surgeons has indicated that COVID-19 has had a profound impact on their outpatient and surgical trauma and non-trauma practice, due to the lockdown and resulted in significant changes to hospital protocols. Preparedness to resume clinical and surgical practice was associated with anxiety in two-thirds of the respondents. Majority of the orthopaedic practitioners felt that they would continue to conduct pre-operative COVID-19 screening and use PPE even after the lockdown is over.

## Introduction

The World health Organisation (WHO) declared severe acute respiratory syndrome coronavirus 2 (SARS-CoV-2) outbreaks as a global pandemic on 11 March 2020^1^. To contain the pandemic, Government of India announced many steps such as nationwide lockdown on 25 March 2020, promoting social distancing, use of masks, personal protective equipment (PPE), setting up of COVID-19 hospitals, quarantine, testing facilities and tracing of contacts. As of 30 May 2020, India has had a total of 1,82,143 confirmed cases and 5,164 confirmed hospital deaths attributed to COVID-19^2^. In India, lockdown has significantly affected the access to health care facilities and disrupted the traditional realms of orthopaedic practice including accessibility and delivery of orthopaedic devices and equipment’s^3,4^. The Indian Orthopaedic Association (IOA) and the British Orthopaedic Association (BOA) have published guidelines on management of patients with urgent orthopaedic conditions and trauma during the coronavirus pandemic^5,6^.

Being mindful of travel restrictions and difficulties of collecting data in the field, an online cross-sectional questionnaire survey was undertaken to evaluate the impact of COVID-19 on orthopaedic practice in India, during the lockdown period from 25 March 2020 to 17 May 2020 and to assess the preparedness of orthopaedic surgeons for clinical practice after lockdown period of the pandemic. It would be prudent to say that as lockdown period has been extended to 31 May 2020, however in this article the readers are advised to consider the lockdown period as from 25 March 2020 to 17 May 2020.

## Material and Method

This cross-sectional study used an online questionnaire of 35 questions. It was developed and circulated through different platforms such as electronic mail and social media smart phone WhatsApp and Facebook applications. The questionnaire was circulated on 17 May 2020 and closed on 24 May 2020 (8 days period) to allow for data interpretation, preparation, and submission of this article to the journal website.

The target audience were orthopaedic surgeons with post graduate qualification in orthopaedics and currently practising in India. Orthopaedic surgeons practising abroad, non-practising surgeons and doctors still pursuing orthopaedic postgraduate training were not included in analysis.

The responses submitted were checked for duplication, pooled, analysed, and summarised. The focus of the survey was on the impact on pattern of orthopaedic practice in hospitals amidst COVID-19, assess development of new orthopaedic surgical protocols in view of pandemic situation, changes in Out-Patient Department (OPD) practice, availability and practices regarding Personal Protective Equipment (PPE) usage and Operation Theatre (OT) set up for infection control, impact on OT procedures and future preparedness for clinical practice, after the lockdown period. Data was entered in SPSS (Statistical product and service solutions) version 16 [IBM Corp] for statistical analysis. Frequencies/percentages were summarised and Chi square test for categorical variables, and Fisher's exact test used as appropriate. Wilcoxon sign rank test was used to compare the patients visiting the outpatient department (OPD) and patients operated this year (amidst COVID-19 pandemic) and last year during the same period specified. P value < 0.05 was considered as significant.

## Results

We sent out questionnaires of 35 questions to approximately 12,000 orthopaedic surgeons from various states and union territories of India, by email and other social media outlets. We received 588 responses Table I. The data was checked for duplication. Majority of respondents were orthopaedic surgeons working in private hospitals (75.1%). Some responses by the orthopaedic surgeons have been tabulated in Table II.

**Table I:** State wise distribution of respondents

S No.	Indian States	No. of respondents
1	Jammu and Kashmir	04
2	Himachal Pradesh	03
3	Punjab	20
4	Haryana	24
5	Delhi	136
6	Uttarakhand	10
7	Uttar Pradesh	71
8	Rajasthan	13
9	Gujarat	36
10	Madhya Pradesh	22
11	Bihar	12
12	Jharkhand	04
13	West Bengal	16
14	Maharashtra	50
15	Chhattisgarh	09
16	Telangana	23
17	Andhra Pradesh	16
18	Karnataka	37
19	Kerala	15
20	Tamilnadu	59
21	Tripura	01
22	Assam	05
23	Odisha	02

**Table II:** Responses by orthopaedic surgeons

S No.	Questions	Response	
1.	Your institute/Hospital is a	Government (24.83%)	Private (75.17%)
2.	Capacity of orthopaedic beds in your hospital?	> 50 beds (54.94%)	< 50 beds (45.06%)
3.	Number of trauma cases performed in your hospital from 25/03/2019 to 17/05/2019.	up to 20 cases (46.43%)	up to 50 cases (21.94%)
4.	Number of trauma cases performed in your hospital from 25/03/2020 to 17/05/2020.	5 or less (34.01%)	10 cases (14.97%)
5.	Orthopaedic OPD cases seen from 25 March 2019 to 17 May 2019 at your Hospital.	1-200 (37.24%)	>200 (62.76%)
6.	Orthopaedic OPD cases seen from 25 March 20120 to 17 May 2020 at your Hospital.	0-50(30.44%)	> 51 (69.56%)
7.	Has your Orthopaedic team been redeployed to perform other duties in the hospital?	No (69.22%)	Yes (30.78%)
8.	Has your Hospital received any known COVID-19 infected patients?	No (57.84%)	Yes (42.16%)

**Fig. 1:**
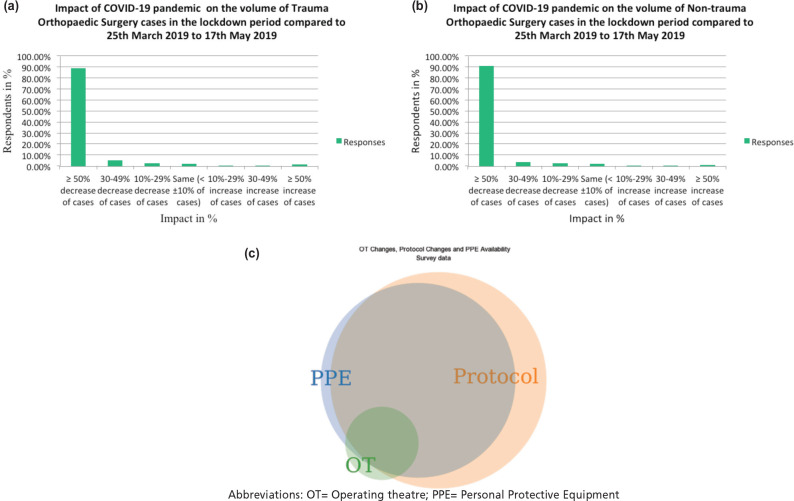
(a) Impact of COVID-19 pandemic on the volume of Trauma Orthopaedic Surgery cases in the lockdown period compared to 25th March 2019 to 17th May 2019. (b) Impact of COVID-19 pandemic on the volume of Non-trauma Orthopaedic Surgery cases in the lockdown period compared to 25th March 2019 to 17th May 2019. (c) Association between OT changes, Hospital protocol changes and PPE’s availability undertaken during COVID-19.

We observed that COVID-19 has had a significant impact on the orthopaedic practices, in India. There was a significant effect noticed on the OPD practice, with a reduction in footfall of patients and further reduction in it, as the lockdown period was extended (p<0.01). Fig. 1(a) and 1(b) show the impact of COVID-19 on the volume of trauma and non-trauma surgery as compared to the corresponding time of 2019. Significant changes were instituted in the surgical protocols of the hospitals (91.7%) Table III. Fig. 1(c) shows the association between OT changes, hospital protocol changes and PPE’s availability undertaken during COVID-19.

**Table III:** Changes instituted in the surgical protocols of hospitals

S No	Changes in surgical protocols
1.	Pre-operative screening of all patients for COVID-19.
2.	Conservative management of fractures, as far as possible (with an acknowledgement of need of delayed reconstruction)
3.	Restricted OPD timings and consultations
4.	Thermal scanning of the patients attending OPD and hospitals
5.	Increased use of PPE, hand sanitizers, and following social distancing principles.
6.	Separate COVID-19 wards and OT facilities.

Surgery for fragility fractures was done by 68.71% respondents during the lockdown; less than five cases were operated by 37.76%, and 5 to 20 cases were operated by 5.44% respondents. Septic arthritis and prosthetic joint infections were operated by 17.01%. Low velocity trauma (59.86%) was the most common cause of injury, during the lockdown period, followed by the road traffic accident (RTA) (40.14%).

**Fig. 2:**
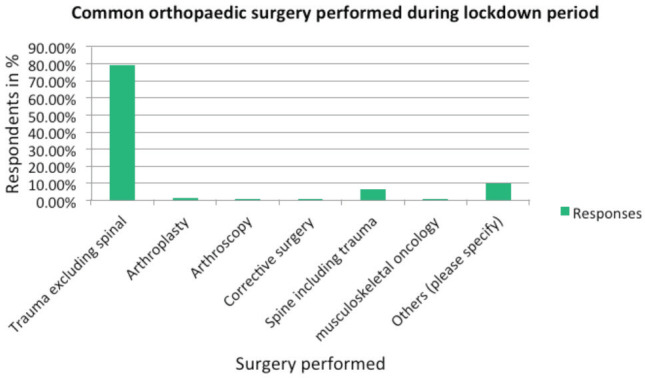
Common orthopaedic surgery performed during lockdown period.

Trauma surgery excluding spinal trauma was the most common orthopaedic surgery performed (79.25%). Apart from this, 6.63% performed spine surgery including trauma and approximately 3.4% surgeons performed arthroscopy, arthroplasty and musculoskeletal oncology surgeries combined (Fig. 2). Majority of the respondents (74.32%) claimed that their hospitals had provision of PPE kits while performing surgeries in COVID-19 negative patients. However, there appeared to be an increased emphasis on the use of recommended PPE by the surgeons, during operative procedures; 68.54% of Orthopaedic surgeons have used PPE while performing surgery and 59.86% surgeons have also used PPE in non-COVID-19 patients. Interestingly, surgeons who were ready to resume traditional surgery after the lockdown seemed more health conscious and would prefer PPE use during surgery even for patients who test negative for COVID-19 (p=0.019, Chi square=5.589). Surgeons who wore PPE for COVID negative cases also strongly felt that a COVID-19 screening should be performed for every operative case after lockdown (p=0.013, Chi square=6.555). Majority of hospitals (68.02%) cancelled elective surgery. There was a significant reduction in the total number of surgical procedures performed in the period, compared to similar period in the previous year (p=0.00). No remodelling to upgrade the existing OT infrastructure was done by the majority (89.48%), but majority were cognisant of the fact that there will be requirement of modifying OT ventilation systems once the elective surgery resumes.

We noted that 63.27% of respondents had plans to install OT filters that are able to remove aerosols and 56.63% preferred a ventilation system in the operating theatre with a minimum of 20 air changes per hour. Moreover, those units which were ready to remodel OTs or open new OTs, were also getting ready for procuring further PPE kits (p-value=0.01, Chi square= 5.928) and for making Protocol Changes (p=0.048, Chi square=4.098).

**Fig. 3:**
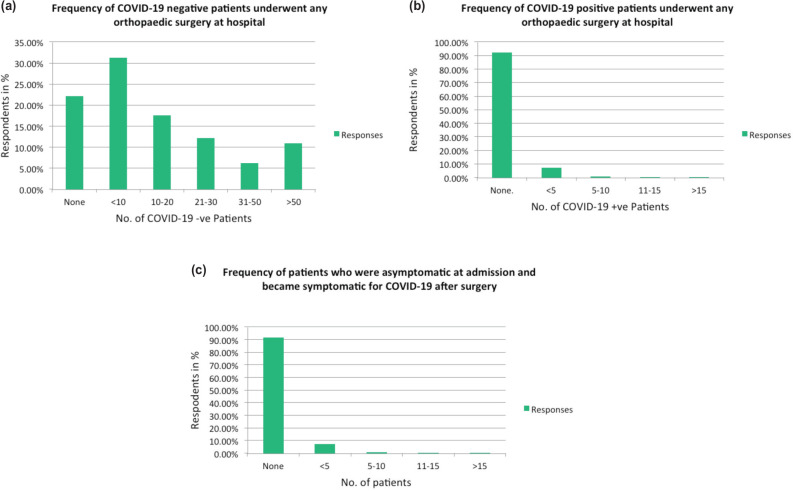
(a) Frequency of COVID-19 positive patients who underwent any orthopaedic surgery at hospitals. Frequency of COVID-19 negative patients who underwent any orthopaedic surgery at hospital. Frequency of patients who were asymptomatic at admission and became symptomatic for COVID-19 after surgery.

A total of 504 surgeons (85.7%) claimed that they had performed surgery during the aforesaid period. Fig. 3(a) and 3(b) shows the frequency of respondents who operated on COVID-19 negative and positive patients, respectively. The intra-operative and post-operative mortality was reported by 18 respondents (3.06%), during lockdown period. Fig. 3(c) shows the assertion of 8.5% surgeons that their patients changed from asymptomatic to symptomatic status for COVID-19, after performing surgery

There was a lot of anxiety (60.88%) associated with the thought of resuming surgery, when the lockdown measures are lifted. However, there was no association between PPE usage (cumbersome to do surgery with PPE donned) among orthopaedic surgeons who reported anxiety (p=0.856, Chi square=0.062). Also, surgeons who were ready to operate patients after the end of lockdown period preferred to use PPE for every case after lockdown, and the association was statistically significant. 69.73% have shown preference to use PPE during every surgery during pandemic after the lockdown is over (p<0.01, Chi square=24.446).

A total of 88.07% felt that the pre-operative screening for COVID-19 be performed for every patient undergoing surgery and 72.28% respondents believed they would not perform surgery without performing testing for COVID-19 (p<0.01, Chi square=24.251).

The five most common responses, as to when the orthopaedic surgeons would think of resuming elective surgery were (a) Government orders to open government hospitals with mandate to perform elective surgery (45.58%), (b) Lockdown in the region has been lifted (35.37%) and (c) The hospital has an adequate supply of PPE and has facility for Reverse Transcriptase – Polymerase Chain Reaction (RT-PCR) test for SARSCoV-2 virus (34.18%) (d) The hospital is able to maintain social distancing measures throughout the pre-operative, intra and post-operative period (31.8%) and (e) There is decrease in the number of COVID-19 cases in the region (29.08%).

With regards to changes in OT protocols, majority believed the following changes would be prerequisite in resuming orthopaedic surgery: (a) limiting the number of people in operating theatre (89.8%), (b) keeping the power setting of tools as low as possible when high-power tools are being used, or considering alternative use of Gigli saw, osteotomes and manual reaming whenever possible (75.85%), (c) judicious use of suction to remove smoke and limiting the use of electrocautery (69.9%).

A change in the post-operative protocol for patients on resumption of elective surgery was stated by majority with 93.03% of the opinion on decreasing the length of hospital stay and follow-up visits to be minimised. This would be facilitated with the help of telemedicine (76.7%) and patient to be instructed on home rehabilitation strategies (69.56%). Tele-health appeared to be the preferred proposed mode of consultation in future for 80.44%.

## Discussion

The COVID-19 pandemic has had a profound effect on health care system and orthopaedic surgeons in India^4,7^. Current literature does highlight experiences of managing orthopaedic and trauma conditions during the pandemic, with some providing guidelines on strategies to resume elective surgery^8-11^. However, there are no reports from Indian sub-continent on the effect of COVID-19 on orthopaedic practice due to the lockdown and directions for resuming future surgery, except for a recent survey assessing the psychological impact of the lockdown during COVID-19 on orthopaedic surgeons^12^. Majority of the responses received were from private practitioners, perhaps because of more available time amid concerns about their private practice and significantly reduced incomes. The effect of COVID-19 on orthopaedic practice in India has been similarly experienced by various allied surgical specialities of organ transplantation, urology, and colorectal surgery^13-15^. Libeinsteiner *et al* reported drastic reduction of arthroscopic and almost total closure of arthroplasty surgery in Austria, Germany, and Switzerland^16^. Thaler *et al* performed an online survey of arthroplasty surgeons in Europe who were members of European Hip Society (EHS) and the European Knee Associates (EKA) to evaluate the impact of the COVID-19 pandemic on joint arthroplasty service in 272 surgeons from 40 countries. The major finding of this study was a drastic reduction in primary and revision arthroplasty surgery^17^. Both studies suggest similar findings to ours, suggesting, despite reduction in elective orthopaedic surgery; surgery for acute fractures and infections continued being performed. Our survey has also noticed severe (>50%) reduction in volume of trauma and non-trauma surgery being performed in lockdown period as compared to the corresponding time of 2019. An inclination towards conservative management of non-obligatory fractures, in the current situation, with an acknowledgement that some of these injuries may require delayed reconstruction in the future has been recognised^18^. This has also rekindled interest in classical and newer techniques of non-surgical treatment of fractures amongst the newer generation of orthopaedic surgeons. Majority of orthopaedic surgeons have cancelled the elective surgery, as advised by the Government of India, to contain the spread of COVID-19 and thus limiting unnecessary exposure of patient and healthcare professionals, conserving the use of PPEs, redistributing the existing infrastructure and re-organising manpower to combat the pandemic. It was apparent from our survey that there was a significant reduction in the number of surgery performed, compared to a similar period in the previous year. Similarly, the drop in patients attending OPD was significant, due to lockdown measures. However, management of obligatory and fragility fractures remained priority even during the lockdown and surgeons strived to operate such cases promptly to discharge them early after an immediate post-operative rehabilitation session^19,20^. It was realised that the incidence of fragility fracture may remain the same during pandemic^21^. Orthopaedic surgeons (69.22%) who have responded in this survey mentioned they have not been redeployed elsewhere in their other hospital for example to other specialities, a feature seen in other global health care systems during the pandemic. The probable reasons being that majority of the respondents were from the private clinics and not from public hospitals. This has allowed them to cater to specialised orthopaedic trauma work for patients requiring emergent treatment. PPE supply and its availability has been a constant concern for all health care workers across the word and those on the coronavirus frontline including orthopaedic surgeons. Guo *et al* reported COVID-19 infection in 24 orthopaedic surgeons in Wuhan City, China during the early course of outbreak. They concluded that lack of exposure of orthopaedic surgeons to deal with contagious disease, insufficient infection control measures, and lack of awareness for the disease which was compounded by the insufficient supply of PPE leads to their infection^22^. Our survey has also demonstrated that most hospitals had provision of use of PPE (74.3%) and 68.54% of orthopaedic surgeons have used PPE while performing surgery whilst 59.86% surgeons have used PPE while performing surgery in patients even if they tested negative for COVID-19 patients. As noticed, all over the world, in the initial stages of the pandemic, the availability did appear to be stretched. This could explain why the compliance of the PPE was not 100%. However, the importance of PPE and infection control strategies was quickly established and accepted following public health guidelines. It is indicative of hospital or surgeon’s awareness and willingness to upgrade their knowledge. It does appear efforts were undertaken by them and supported by the health care system to protect both the patients and staff from contracting COVID-19 infection.

Majority of orthopaedic surgeons surveyed did not operate on COVID-19 patients (92.18%) and 77.89% surgeons operated on COVID-19 negative patients. Understandably, there was concern amongst surgeons about treating patients who presented with COVID-19 disease or would develop infection in the post-operative period. Mi *et al* reported 10 patients with COVID-19 infection and fractures and seven of these patients contracted COVID-19 as nosocomial infection; and four died without surgery being performed. They all had leucocytosis with lymphopenia^23^. They thus proposed that non-operative treatment should be employed as far as possible, strict infection control measures should be in place for patients, patients should be monitored and treated more aggressively and surgery should be performed in negative pressure operation theatre. These findings can be explained on the basis that the surgeons are aware of the consequences or requisite OT infrastructure or good ICU facilities were lacking. The respondents who did not make changes to the existing infrastructure of their operation theatres were 89.46%. It was perhaps due to the financial implications, lockdown situation and non-surety about its future implications and were therefore fearful or hesitant of performing surgery on COVID-19 patients in their present OT set ups.

A total of 8.5% surgeons claimed that patients at their hospitals converted from asymptomatic to symptomatic status for COVID-19 after performing surgery. Rabie *et al* do report seven cases of diagnosed COVID-19 infection in orthopaedic patients presenting with fractures, out of these three patients were asymptomatic for COVID-19 at the time of presentation and two became symptomatic next day of surgery^24^. They suggested prodromal fatigue and tiredness might have pre-disposed these patients to the fall, with evidence appearing in the early post-operative period after COVID-19 tests were performed. Intra and post-operative mortality was reported by 18 respondents (3.06%) during lockdown period. Traditionally, after the orthopaedic surgery, mortality follows established figures, except in case of high velocity trauma. However significant trauma injuries were very much reduced during the lockdown period and predominantly elderly patients with comorbidities underwent surgery.

Recently study from COVID Surg Collaborative group suggests perioperative SARS-CoV-2 infection are associated with high mortality^25^. Similarly, Maniscalco *et al* in their study reported 17 deaths in patients in 121 patients with proximal femur fracture compared to corresponding time of 2019^26^. We suggest that the orthopaedic surgeons should keep a high threshold for surgery during the COVID-19 pandemic, particularly in men aged 65 years and older. It is preferred to postpone non-urgent procedures and promoting non-operative treatment to delay or avoid the need for surgery^25^.

Knowledge of COVID-19 has evolved around strategies to prepare for resuming future surgery. Majority (88.07%) feel pre-operative screening for COVID should be performed for every patient undergoing surgery and they (72.28%) would not perform surgery without performing testing for COVID-19. 69.73% have shown preference to use PPE during every surgery even after the lockdown is over^11^. The cause of anxiety in 60.88% cases of resuming surgery post lockdown could be due to several reasons e.g., a) social stigma associated with COVID-19 infection, b) fear of high complication rate, c) fear of infection spreading to self and to colleagues, d) apprehension regarding continued supply and availability of PPE for prolonged periods after lockdown.

This study has some limitations, due to its inherent nature. It attracted a low response rate from a subset of practising orthopaedic surgeons belonging to a vast country with approximately 12,000 orthopaedic surgeons^27^. Still, we feel that the numbers of respondents are adequate enough to derive useful conclusions. A recently published COVID-19 survey from large population bases have had similar response rates including 610 responses in recent study of impact of COVID-19 on mental health of Indian Orthopaedic surgeons^12,28^. Perhaps other people who were sent the survey did not have time or interest to respond due to stretched resources, personal anxieties, stress and undertaking patient care in unprecedented set of circumstances^12^.

We feel that there are some strengths associated with this study. Firstly, such a cross-sectional survey of orthopaedic surgeons from across the country pertaining to several relevant factors related to their clinical practice has ever been performed or reported from India. Secondly, this survey has brought in many important findings, which shall provide guidance to the orthopaedic surgeons when they resume their practices soon.

## Conclusion

This pan India survey of Indian orthopaedic surgeons has confirmed that COVID-19 has had a profound impact on their trauma and non-trauma practice, with severe reduction in the numbers of outpatient attendance and Orthopaedic surgical procedures, compared to similar time last year. The non-emergency surgery were cancelled by the most surgeons and the conservative management was preferred as far as possible. Only the obligatory low velocity fractures (like proximal hip fractures in elderly) and severe RTA cases were operated during this pandemic. Significant changes were made in the individual hospital protocols (91.7%), like the use of masks, gloves, and PPE, frequent sanitisation of hand and the hospital premises, maintaining social distancing and pre-operative COVID-19 testing of the patients. The use of PPE in COVID-19 positive cases was a norm, but its use in the COVID-19 negative cases was not universal. The remodelling or upgrading of the operation theatres was however not done by the most hospital during the pandemic, as discussed before for several possible reasons.

The majority of surgeons did not perform orthopaedic surgery on COVID positive cases. The post-operative mortality was 3.06%, which is reasonably high from orthopaedic surgery point of view. The preparedness to resume clinical and surgical practice was associated with anxiety in around two-thirds of the respondents. The majority felt that they would continue to do pre-operative COVID-19 screening and use PPE after the lockdown is over. Respondents were keen to limit the number of OT personnel, avoid the use of Aerosol Generating Procedures, minimise the length of stay of the patients and use tele-health for the follow-up of their patients, as far as possible.
